# Accurate classification of membrane protein types based on sequence and evolutionary information using deep learning

**DOI:** 10.1186/s12859-019-3275-6

**Published:** 2019-12-24

**Authors:** Lei Guo, Shunfang Wang, Mingyuan Li, Zicheng Cao

**Affiliations:** 1 Department of Computer Science and Engineering, School of Information Science and Engineering, Yunnan University, Kunming, 650504 People’s Republic of China; 2 School of Public Health (Shenzhen), Sun Yat-sen University, Guangzhou, 510006 People’s Republic of China

**Keywords:** Deep learning, Membrane protein type prediction, Vector representation

## Abstract

**Background:**

Membrane proteins play an important role in the life activities of organisms. Knowing membrane protein types provides clues for understanding the structure and function of proteins. Though various computational methods for predicting membrane protein types have been developed, the results still do not meet the expectations of researchers.

**Results:**

We propose two deep learning models to process sequence information and evolutionary information, respectively. Both models obtained better results than traditional machine learning models. Furthermore, to improve the performance of the sequence information model, we also provide a new vector representation method to replace the one-hot encoding, whose overall success rate improved by 3.81% and 6.55% on two datasets. Finally, a more effective model is obtained by fusing the above two models, whose overall success rate reached 95.68% and 92.98% on two datasets.

**Conclusion:**

The final experimental results show that our method is more effective than existing methods for predicting membrane protein types, which can help laboratory researchers to identify the type of novel membrane proteins.

## Background

Protein is an important component of all cells and tissues in the human body and is the material basis of life [1–5]. Membrane proteins represent one important protein type that is rich in function, they participate in many important reactions of the cell, including transporting substances into and out of cells as a carrier, acting as a specific receptor for hromones, and carrying out cellular recognition functions, as well as being responsible for signal transduction and cell-cell interactions [6]. Among the genomes that have been completely sequenced, the number of membrane proteins accounts for 30% [7]. Moreover, membrane proteins are of particular importance in drug therapy, as they act as the targets for many drugs. Currently, the effects more than 50% of drugs on the market are exerted by the actions of membrane proteins [8]. Since the function of membrane protein is closely related to its type, the prediction of membrane protein types can contribute to research in the field of bioinformatics.

According to their functions, membrane proteins can be classified into three classes: integral, peripheral and lipid-anchored [9]. Based on the direct interaction relationship between membrane proteins and lipid bilayers, the three classes can be further extended into eight basic types: (1) type I membrane proteins, (2) type II membrane proteins, (3) type III membrane proteins (4) type IV membrane proteins, (5) multipass transmembrane proteins, (6) lipid chain-anchored membrane proteins, (7) GPI-anchored membrane proteins, and (8) peripheral membrane proteins. Among them, Types I, II, III and IV are single-pass transmembrane proteins, and detailed descriptions of their differences are given in [9].

To help laboratory researchers discover the type of novel membrane protein, various computation methods are proposed for membrane protein type recognition. Many of these approaches incorporate machine learning algorithms and statistical analysis techniques, such as k-nearest neighbor (KNN) [10], the naive Bayesian model (NBM) [11], support vector machines (SVM) [12–14], random forests (RF) [15], probabilistic neural network (PNN) [16] and hidden Markov models [7].

The most popular feature extraction methods for predicting membrane protein types are based on sequence information. The pseudo-amino acid composition (PseAAC) [17] method incorporates information about sequence order. Local amino acid composition (LAAC), local dipeptide composition (LDC), global descriptor (GD), Lempel-Ziv complexity (LZC), autocorrelation descriptor (AD), sequence-order descriptor (SD) and Hilbert-Huang transform (HHT) are proposed based on amino acid classification and physicochemical properties [18], and these methods are partially applied in our previous research [19]. The peptide composition method, such as dipeptide composition (DipC) [20] and tripeptide composition (TipC) [21] are also powerful sequence information-based feature extraction methods.

The evolutionary information, which mainly refers to the position-specific scoring matrix (PSSM), is also widely used to predict membrane protein types [20, 22]. To extract rich evolutionary information from PSSM, many researchers have proposed their own methods, such as the reduced position-specific score matrix (RPSSM) [23], evolutionary difference position-specific score matrix (EDPSSM) [24], tri-gram position-specific score matrix (TriPSSM) [25], k-separated-bigrams position-specific score matrix (KPSSM) [26], correlation position-specific scoring matrix (CoPSSM) [27], and pseudo position-specific score matrix (PsePSSM) [9]. These methods are all obtained through complex feature engineering.

In this paper, we improve the performance in membrane protein type prediction. The main contributions of the paper are summarized as follows: First, we present two deep neural network (DNN) models to process the sequence information and evolutionary information separately. These models both achieve better performance than traditional machine learning models. Second, by using the DNN model with convolutional and recurrent layers, we can remove the burden of feature engineering and the reliance on domain experts. Third, we provide a new vector representation based on autoencoder and physicochemical property indexes. Numerous experimental results prove that the new vector has more powerful representation ability than one-hot encoding. Finally, a more effective model is constructed by fusing the above two models with the ensemble learning.

The DNN model has shown its superiority in the field of bioinformatics [28–37]. However, to the best of our knowledge, this paper is the first to propose DNN models for the prediction of membrane protein types. The two DNN models proposed in this paper can process the sequence information and evolutionary information, separately. When processing sequence information, we use a 1D convolutional layer [38] and bidirectional long short term-memory (Bi-LSTM) layers [39], and when processing evolutionary information, a 2D convolutional layer and CapsNet layer [40] are adopted. Among these layers, compared to convolutional network, CapsNet is a new network that can extract local position information.

## Methods

### Datasets

In this work, we use two datasets to evaluate the performance of our method, hereafter referred to as Dataset 1 and Dataset 2. Dataset 1 consists of two benchmark sets, the training set and the testing set, which are both from paper [41] and used in previous studies [9, 10, 12, 13, 20]. Dataset 2 is constructed by Chen [12]; it also consists of two benchmark sets. Table 1 shows the concrete distribution of Dataset 1 and Dataset 2.

**Table 1:** The distribution of samples for Dataset 1 and Dataset 2

Membrane protein types	Dataset 1		Dataset 2	
	Training set	Testing set	Training set	Testing set
Type I	610	444	561	245
Type II	312	78	316	79
Type III	24	6	32	9
Type IV	44	12	65	17
Mutipass	1316	3265	1119	2478
Lipid-chain-anchored	151	38	142	36
GPI-anchored	182	46	164	41
Peripheral	610	444	674	699
overall	3249	4333	3073	3604

### Architecture of the proposed DNN

To improve the performance of the prediction of membrane protein types, we build the DNN model with a keras framework (http://www.keras.io). Considering the respective characteristics of sequence information and evolutionary information, we build two different models to process them, which are hereafter referred to as the sequence information model and evolutionary information model, respectively.

#### Sequence information model

In this model, the input sequences are converted into numerical vectors of fixed length by truncating the long sequence and padding the short sequence with ‘0’. The length of the sequence ranges from 50 to 5000 in our dataset. Although the length of the sequence varies greatly, sequences with a length of less than 1500 account for almost 98% of the total dataset. Therefore, taking the utilization of information and the computational complexity into account, we decided to set the fixed length to 1500. Each of the basic 20 amino acids is converted into a number of 0-19. Ultimately, to prevent different encoding from affecting the performance of model, one-hot encoding is used before inputting the vector into the model.

**Fig. 1:**
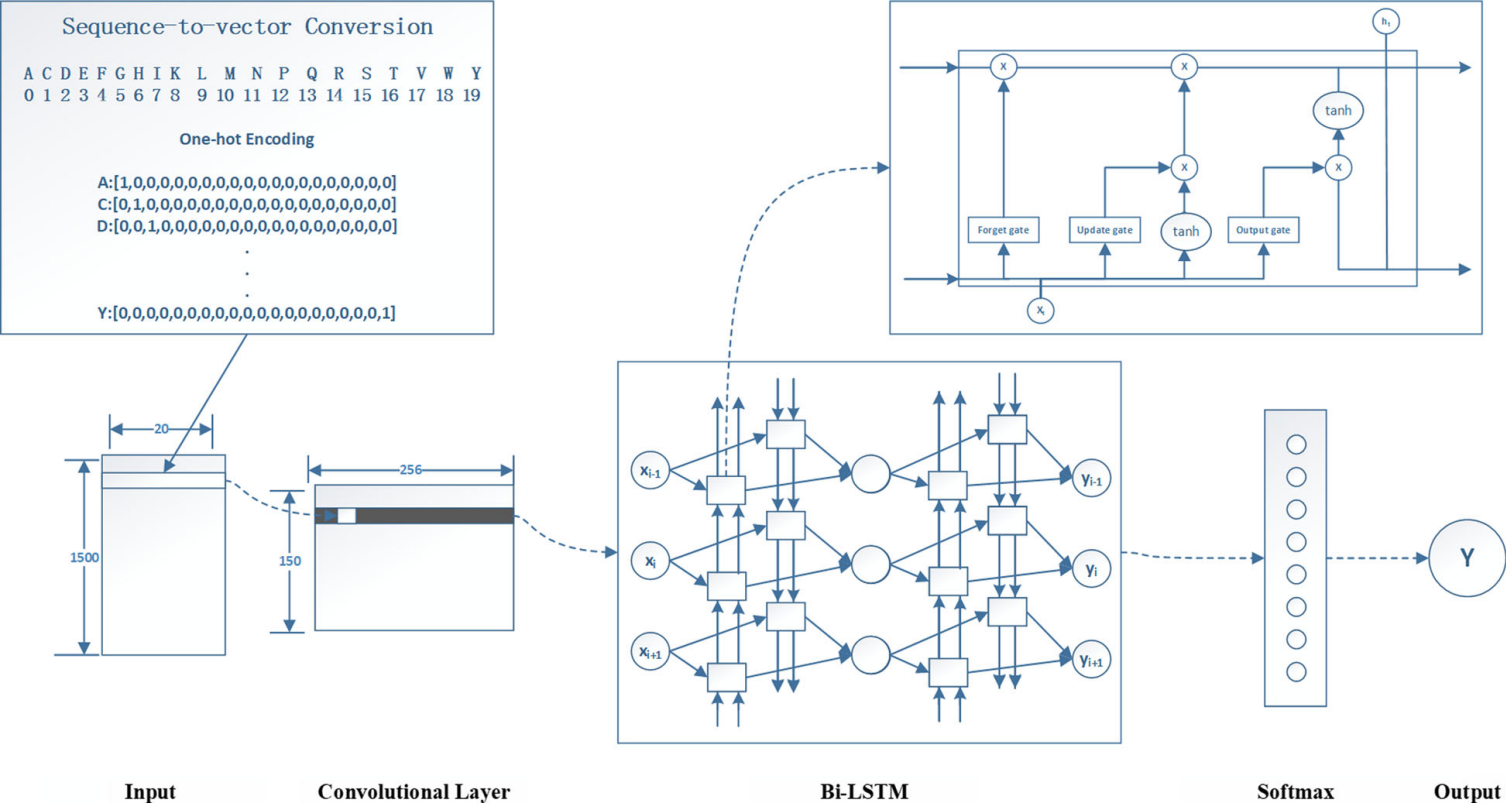
The sequence information model uses 1D Conv and Bi-LSTM layers. First, the membrane protein sequences are encoded into a matrix of size 1500 ×20. Then, these matrixes are fed to the 1D Conv layer (filters: 256, kernel size: 15, strides: 10, padding: same, activation: relu). Next, two Bi-LSTM layers (units: 128, dropout: 0.5 and the remainder as default settings) are used to identify features separated by large gaps. Last, the output from the Bi-LSTM layer is passed through a final dense layer that uses a softmax function to obtain the probability value belonging to each membrane protein type

The architecture of the sequence information model, as shown in Fig. 1, consists of one 1D convolutional layer (filters: 256, kernel__size: 15, strides: 10, padding: ‘same’, activation: ‘relu’), two Bi-LSTM layers (units: 128, dropout: 0.5 and rest default setting) and one fully connected layer (softmax). The 1D convolutional layer can reduce the complexity of the Bi-LSTM layer that reduces the shape of the output matrix from 1500 ×256 tensor to 150 ×256. Moreover, the 256 filters in the layer can also extract rich information from the sequence. Next, two Bi-LSTM layers are applied, which can identify features separated by large gaps. In previous studies, a normal LSTM layer is mainly used to process sequence information [29, 30]. Compared to normal LSTM, the Bi-LSTM layer we used can take advantage of both historical input and future information, which performs better for our task. Each Bi-LSTM unit comprises three gates, which are shown in Fig. 1. Their calculation process is as follows:

1 $$ f_{t}=\sigma (W_{f}\cdot [h_{t-1},x_{t}]+b_{f})\qquad\text{forget\ gate}  $$

2 $$ i_{t}=\sigma (W_{i}\cdot [h_{t-1},x_{t}]+b_{i})\qquad\text{input\ gate}  $$

3 $$ \tilde{C}_{t}=tanh(W_{C}\cdot [h_{t-1},x_{t}]+b_{C})\qquad\text{candidate\ cell\ states}  $$

4 $$ C_{t}=f_{t}*C_{t-1}+i_{t}*\tilde{C}_{t}\qquad\text{updated\ cell\ states}  $$

5 $$ o_{t}=\sigma (W_{o}\cdot [h_{t-1},x_{t}]+b_{o})\qquad\text{output\ gate}  $$

6 $$ h_{t}=o_{t}*tanh(C_{t}))\qquad\text{hidden\ cell\ states}  $$

where *W*_*f*_, *W*_*i*_, *W*_*C*_ and *W*_*o*_ denote the weight matrix of the forget gate, input gate, candidate cell states and output gate, respectively, and *b*_*f*_, *b*_*i*_, *b*_*C*_, and *b*_*o*_ denote the bias of the forget gate, input gate, candidate cell states and output gate, respectively. The forget gate decides which information is discarded from the cell state, where *h*_*t*−1_ represents the output of the previous cell, and *x*_*t*_ represents the input of the current cell. *σ* represents the sigmod function. The input gate decides how much new information is added to the cell states, and it uses two steps to accomplish this task: First, a sigmoid layer determines which information needs to be updated; a tanh layer generates a vector $\tilde {C}_{t}$, which is the alternative content to update. Then, we combine two parts to update the state of the cell. The output gate will determine what value to output. First, we run a sigmoid layer to determine which part of the cell state will be output. Next, we process the cell state through *tanh* (obtaining a value between -1 and 1) and multiply it by the output of the sigmoid gate. Eventually, we will only output the part of our output that we determined.

Then, the output from Bi-LSTM layer is passed through a final dense layer that used a softmax function to obtain the probability value belonging to each membrane protein type. We compile our keras model with the ‘Adam’ optimizer (lr: 0.001, beta__1: 0.9, beta__2: 0.999, decay=0). Finally, we find that adding the BatchNormalization layers (default setting) after three mainly layers in our model helps to accelerate convergence.

#### Evolutionary information model

In this paper, evolutionary information mainly refers to the PSSM, which was first proposed by Jones for predicting the secondary structure of proteins [42]. The PSSM can discover protein sequences that have evolved relationships with search sequences. It is expressed as follows:

7 $$ PSSM= \left[\begin{array}{cccc} M_{1\rightarrow 1} &M_{1\rightarrow 2} &\cdots &M_{1\rightarrow 20} \\ M_{2\rightarrow 1} &M_{2\rightarrow 2} &\cdots &M_{2\rightarrow 20} \\ \vdots &\vdots &\ddots &\vdots \\ M_{L\rightarrow 1} &M_{L\rightarrow 2} &\cdots &M_{L\rightarrow 20} \end{array}\right]  $$

where *L* is the length of the sequence, and *M*_*i*→*j*_ denotes the score of the amino acid in the *i*-th position of the amino acid being mutated to *j*-th position of the amino acid during the evolution process. Because membrane protein sequences vary in size, their PSSM shapes are also different. Similar to the sequence information model, we also truncate the long sequence and pad the short sequence with ‘0’ to convert the PSSM matrix into a 1500 ×20 matrix.

**Fig. 2:**
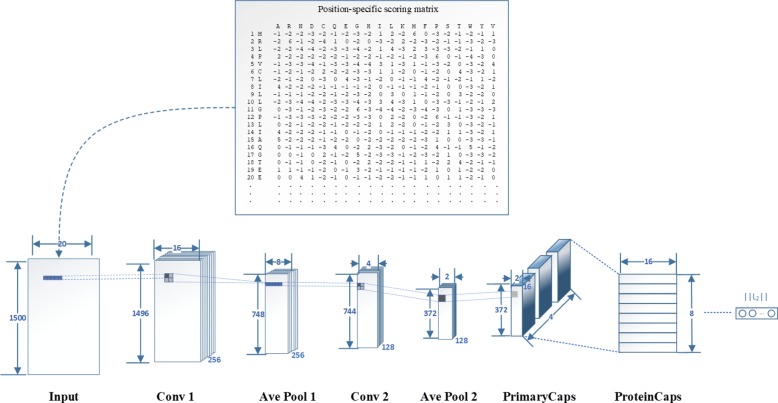
The evolutionary information model uses 2D Conv and CapsNet layers. First, the PSSM matrix is processed as the matrix with the same shape. These matrixes are fed to the 2D Conv layer (filters: 256, kernel size: (5,5), strides: 1, padding: valid, activation: relu) and average pooling layer (pool size: (2,2) and the remainder as default settings) to extract the rich and effective features. Next, the 2D Conv layer(filters: 128, kernel__size: (5,5), strides: 1, padding: ’valid’, activation: ’relu’) and average pooling layer(pool__size: (2,2)) are reused to extract more advanced features. The PrimaryCaps layer is the convolutional capsule layer, which has size 1 ×1 convolution kernels and 4 channels of 16D capsules. The ProteinCaps layer has eight 16D capsules to represent one of membrane protein types. Finally, the L2-norm of each capsule vector is calculated to indicate the probability of each type

The architecture of evolutionary information model is shown in Fig. 2. It consists of two 2D convolutional layers, two average pooling layers, one PrimaryCaps and one fully connected layer. The first 2D convolutional layer (filters: 256, kernel__size: (5,5), strides: 1, padding: ‘valid’, activation: ‘relu’) is used to extract rich and effective features from the PSSM. Next, the first 2D average pooling layer (pool__size: (2,2) and the remainder as default settings) is used to retain the main features while reducing the parameters and calculations of the next layer to prevent overfitting. Although paper [40] argued that the pooling layer may throw away information about the precise position of the entity within the region, we find that the pooling layer and CapsNet work together to obtain the best results in our task. One possible reason is that the pooling layer can improve the robustness of extracted features. Then, the second 2D convolutional layers (filters: 128, kernel__size: (5,5), strides: 1, padding: ‘valid’, activation: ‘relu’) and the second 2D average pooling layer (pool__size: (2,2) and the remainder as default settings) are reused to obtain more advanced features. Moreover, each convolutional layer has a dropout technique with the rate 0.5 to prevent the model from overfitting. The first four layers are designed to increase the representation power of CapsNet.

The PrimaryCaps layer contains 4 primary capsules that accept the basic features detected by the first four layers and generate a combination of features. The 4 main capsules of this layer are essentially similar to the convolutional layer. Among them, the 1 ×1 convolution kernel used in our study not only achieves cross-channel interaction and information integration, but also reduces the number of convolution kernel channels. Each capsule applies sixteen 1 ×1×64 convolution kernels to the 372 ×2×128 input tensor, thus generating a 372 ×2×64 output tensor. With a total of 4 capsules, the output is a 372 ×2×4×16 tensor. Since the length of a capsule represents the probability that the entity presented, the CapsNet layer needs a new activation function, which is called the squashing function. It is a novel nonlinear activation function that accepts an input vector and then compresses its length to [0,1] as output, which is calculated as follows:

8 $$ V_{j}=\frac{\left \| s_{j} \right \|}{1+\left \| s_{j} \right \|^{2}}\frac{s_{j}}{\left \| s_{j} \right \|}  $$

where *V*_*j*_ and *s*_*j*_ are the vector output and input of the CapsNet, respectively.

**Fig. 3:**
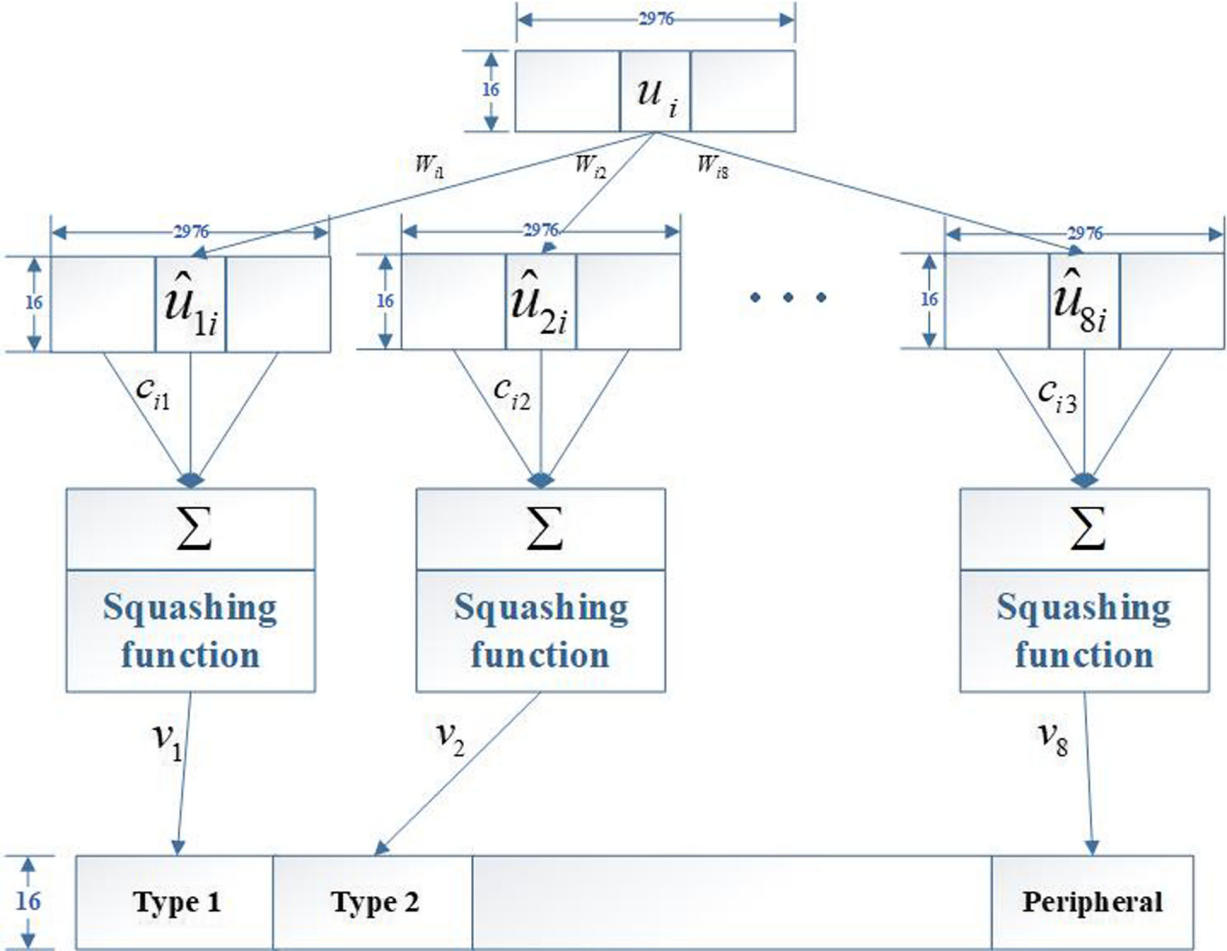
Computation between the PrimaryCaps and ProteinCaps layers.There are 2976 16D capsules (each *u*_*i*_ is an 16D vector) in PrimaryCaps. Each $\hat {u_{j|i}}$ is produced by multiplying *u*_*i*_ by a weight matrix *W*_*i*,*j*_ (16 ×16). Capsule *V*_*j*_ (16D vector and *j*∈ [1, 8]) in ProteinCaps is produced by a weighted sum over all $\hat {u_{j|i}}$ and the squashing nonlinear activation function. The parameter *c*_*i*,*j*_ is determined by the iterative dynamic routing process

The last layer contains eight membrane protein type capsules, one for each type of membrane protein. The calculation between the PrimaryCaps layer and ProteinCaps layer is shown in Fig. 3. Among them, *u*_*i*_ represents the *i*-th capsule in PrimaryCaps. *W*_*ij*_ is the weight matrix that represents the spatial relationship and other important relationships between low-level capsules and high-level capsules. There are eight capsules (*V*_*j*_,*j*∈[1,2,⋯,8]) in ProteinCaps, each of which receives the inputs from all capsule outputs in PrimaryCaps. *V*_*j*_ is calculated by the weighted sum of $\tilde {u}_{ji}$ and then is passed through the squashing function. Here, the weights *c*_*ij*_ are obtained by an iterative dynamic routing process that can be found in [40].

The output of the ProteinCaps layer has eight 16D vectors. During training, for each training sample, the loss value is calculated as follows:

9 $$ L_{c}\,=\,T_{c}\text{max}(0,m^{+}\!-\left \| V_{c} \right \|))^{2}+\lambda (1-T_{c})\text{max}(0,\left \| V_{c} \right \|)-m^{-})^{2}  $$

where the value of *T*_*c*_ is decided by the correct label. If the correct label corresponds to the membrane protein type in ProteinCaps, then *T*_*c*_=1; otherwise, *T*_*c*_=0. For the value of the other hyperparameters *m*^+^, *m*^−^ and *λ*, we used the default values 0.9,0.1 and 0.5, respectively, which are suggested in [40].Then, eight loss values are added to obtain the final loss.

Finally, we use the ‘Adam’ optimizer method (lr: 0.0001, beta__1: 0.9, beta__2: 0.999, decay=0). The suggested settings, three routing iterations and the margin loss function, are used in our work.

#### Fusion information model

**Fig. 4:**
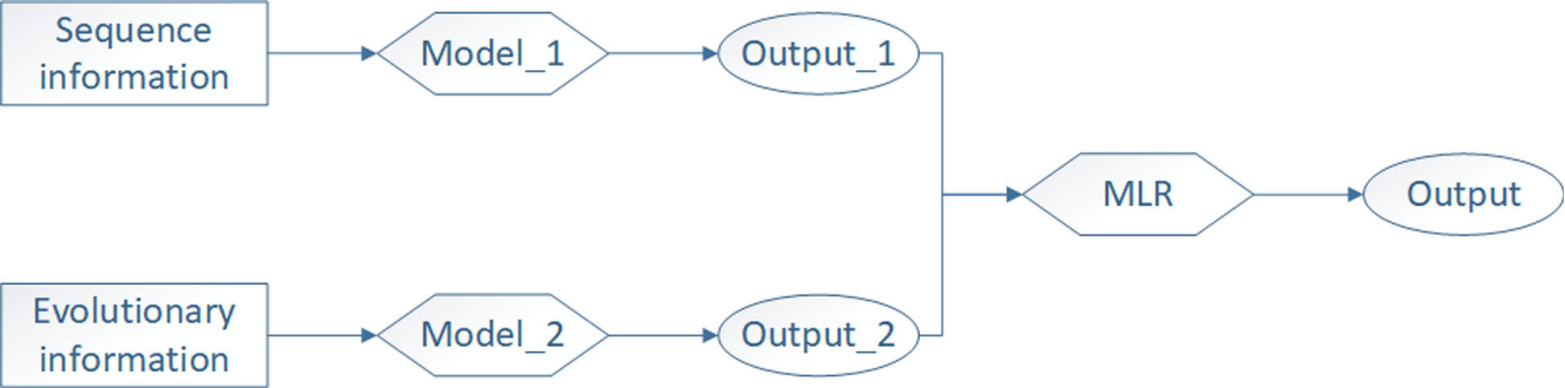
The flow of the stacking algorithm. Model_1 refers to the sequence information model, and Model_2 refers to the evolutionary information model. The class probability outputs of two models are combined to serve as a new training set to train the meta-classifier (MLR). When testing unknown membrane protein samples, we take the result of the meta-classifier as the final output

Many studies have proved that the fusing information method has better results than the single information method [12, 20, 43–45]. Therefore, we also present a fusion information model based on ensemble learning. Among these methods, the ensemble learning method that we adopted is a stacking algorithm, and its details can be found in our previous work [19]. Figure 4 shows the complete flow chart of the stacking algorithm, where the meta-classifier that we used is multiple logistic regression (MLR).

Research has shown that using the class probability output of the base classifier as the input of the meta classifier performs better [46]. Then, we use *P*(*x*) as the output of the base classifier, it can be represented as follows:

$${\begin{aligned} P(x)&=\left(P^{\mathrm{T}}_{1}, P^{\mathrm{T}}_{2}\right) \\ &= \left(\underbrace{P^{1}_{1}, P^{1}_{2}, P^{1}_{3}, P^{1}_{4}, P^{1}_{5}, P^{1}_{6}, P^{1}_{7}, P^{1}_{8}}_{{Sequence\_informtaion\_model}}, \underbrace{P^{2}_{1}, P^{2}_{2}, P^{2}_{3}, P^{2}_{4}, P^{2}_{5}, P^{2}_{6}, P^{2}_{7}, P^{2}_{8}}_{Evolutionary\_informtaion\_model}\right) \end{aligned}} $$

### Model training

In most experiments, the DNN models are trained using identical training strategies. The dataset is divided into three parts, the training set, validation set and testing set, which play different roles in our work. The training set is used to train the model, and the validation set is used to adjust parameters and select the best model. The testing set is used to evaluate the performance of the model at the end. In our work, 20% of samples are separated from the original training set as a validation set. Then, the distributions of three sets in two datasets are shown in Table 2. All DNN models are implemented using keras 2.1.2. Model training and testing are performed on a personal computer equipped with an Nvidia GTX 1060 GPU.

**Table 2:** The distribution of three data in two datasets

Membrane protein types	Dataset 1			Dataset 2		
	Training set	Validation set	Testing set	Training set	Validation set	Testing set
Type I	488	122	444	449	112	245
Type II	250	62	78	253	63	79
Type III	19	5	6	25	7	9
Type IV	35	9	12	52	13	17
Mutipass	1052	264	3265	895	224	2478
Lipid-chain-anchored	121	30	38	114	28	36
GPI-anchored	146	36	46	131	33	41
Peripheral	488	122	444	539	135	699
overall	2599	650	4333	2458	615	3604

### Model evaluation

We use sensitivity (Se), specificity (Sp), accuracy (ACC) and overall success rate (OSR) to evaluate the classification performance. They are defined as follows:

10 $$ Se_{i}=TP_{i}/(TP_{i}+FN_{i})  $$

11 $$ Sp_{i}=TN_{i}/(TN_{i}+FP_{i})  $$

12 $$ ACC_{i}=\frac{TP_{i}+TN_{i}}{TP_{i}+TN_{i}+FN_{i}+FP_{i}}  $$

13 $$ OSR=\frac{\sum_{i=1}^{k}TP_{i}}{\sum_{i=1}^{k}(TP_{i}+FN_{i})}  $$

where *TP*, *TN*, *FP* and *FN* represent true positive, true negative, false positive and false negative, respectively.

The Matthews correlation coefficient (MCC) is also used in our work. It is generally considered to be a more balanced indicator, even if the sample content of the two categories varies widely [47]. The MCC is essentially a correlation coefficient between the actual classification and the predicted classification. Its value range is [-1, 1]. A value of 1 indicates a perfect prediction for the subject. A value of 0 indicates that the predicted result is not as good as the result of random prediction, and -1 means that the predicted classification is completely inconsistent with the actual classification. The MCC is defined as follows:

14 $$ {\begin{aligned} MCC_{i}=\frac{TP_{i}\times TN_{i}-FP_{i} \times FN_{i}}{\sqrt{(TP_{i}+FN_{i})(TP_{i}+FP_{i})(TN_{i}+FP_{i})(TN_{i}+FN_{i})}} \end{aligned}}  $$

### The construction of vector representation

One-hot encoding ignores the possible relationship between amino acids. For example, alanine and cysteine have a certain relationship in their physicochemical properties, yet ‘0’ and ‘1’ do not provide much informtaion about this relationship. Moreover, we usually need more data to train because the amino acids are stored as sparse matrixes. Using a vector representation method can effectively solve the above problems. Continuous bag of words (CBOW) and Skim-Gram [48] are two popular vector representation methods that are widely used in the field of natural language processing (NLP). Limited by the size of protein samples and the characteristics of the protein sequence, these methods are no longer applicable. In this paper, we provide a new vector representation method based on autoencoder and the physicochemical properties of amino acids. This method can achieve a more compact representation of input symbols and yield semantically similar symbols close to each other in vector space.

The AAindex is a database of numerical indexes representing various physicochemical properties of amino acids and pairs of amino acids [49]. The database now has a total of 557 indexes, which all come from the published paper. In this research, the physicochemical properties indexes with ‘NA’ are screened out, and the remaining 537 indexes are adopted. Using a physicochemical properties index that represents the amino acids is a good idea to replace the one-hot encoding. However, this method would consume a large amount of storage space and greatly increase the computational complexity. To process the above problems, autoencoder is used to create a more effective representation.

**Fig. 5:**
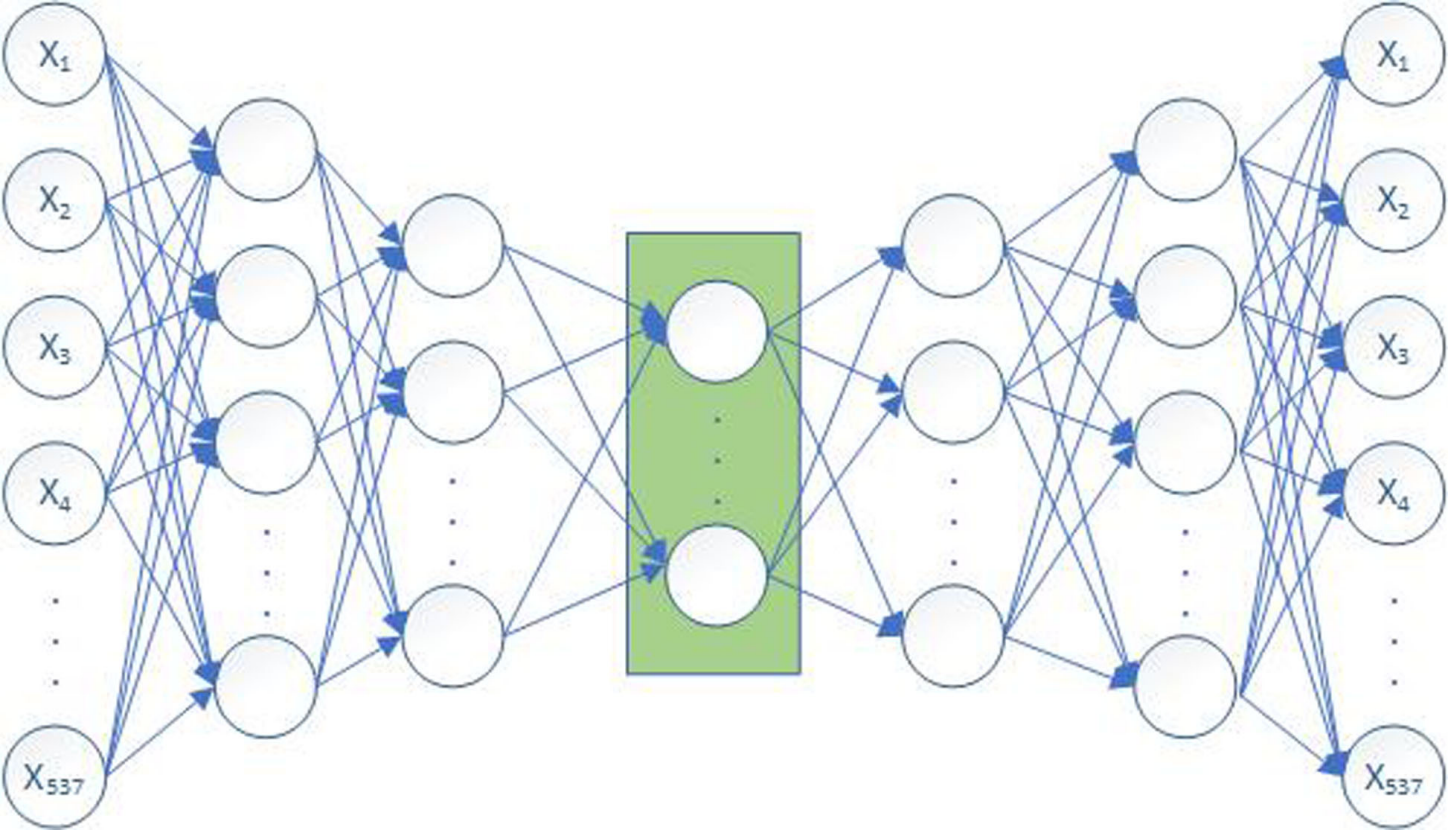
The structure of autoencoder. It consists of an input layer, five hidden layers and an output layer. The input layer has 537 units; the first two layers and the last two layers of the five hidden layers have 128, 64, 64, and 128 units, respectively, the number of nodes in the middle layer of the hidden layers is an additional hyperparameter obtained by training; and the output layer has 537 units

Autoencoder is a neural network that can capture the most important features of data. It consists of the input layer, multiple hidden layers and output layer. The input and output of the autoencoder are consistent, and the goal is to reconstruct itself using some high-level feature recombination. If the number of nodes of the hidden layer is smaller than the input layer and the output layer, it represents the same low-density information and is a centralized representation of the input data obtained from the learning. In our work, we take 537 physicochemical properties indexes of each amino acid as input, reconstruct this informatin with the autoencoder, and then obtain a more effective vector representation from the intermediate layer. The architecture of the autoencoder that we used is shown in Fig. 5.

## Results and discussion

### The performance of vector representation

To explore the advantage of the new vector representation method, we compared the performance between the one-hot encoding and our method in the sequence information model. We note that our process of parameter adjustment is all on the validation set and not reparameterized to apply on the testing set.

**Fig. 6:**
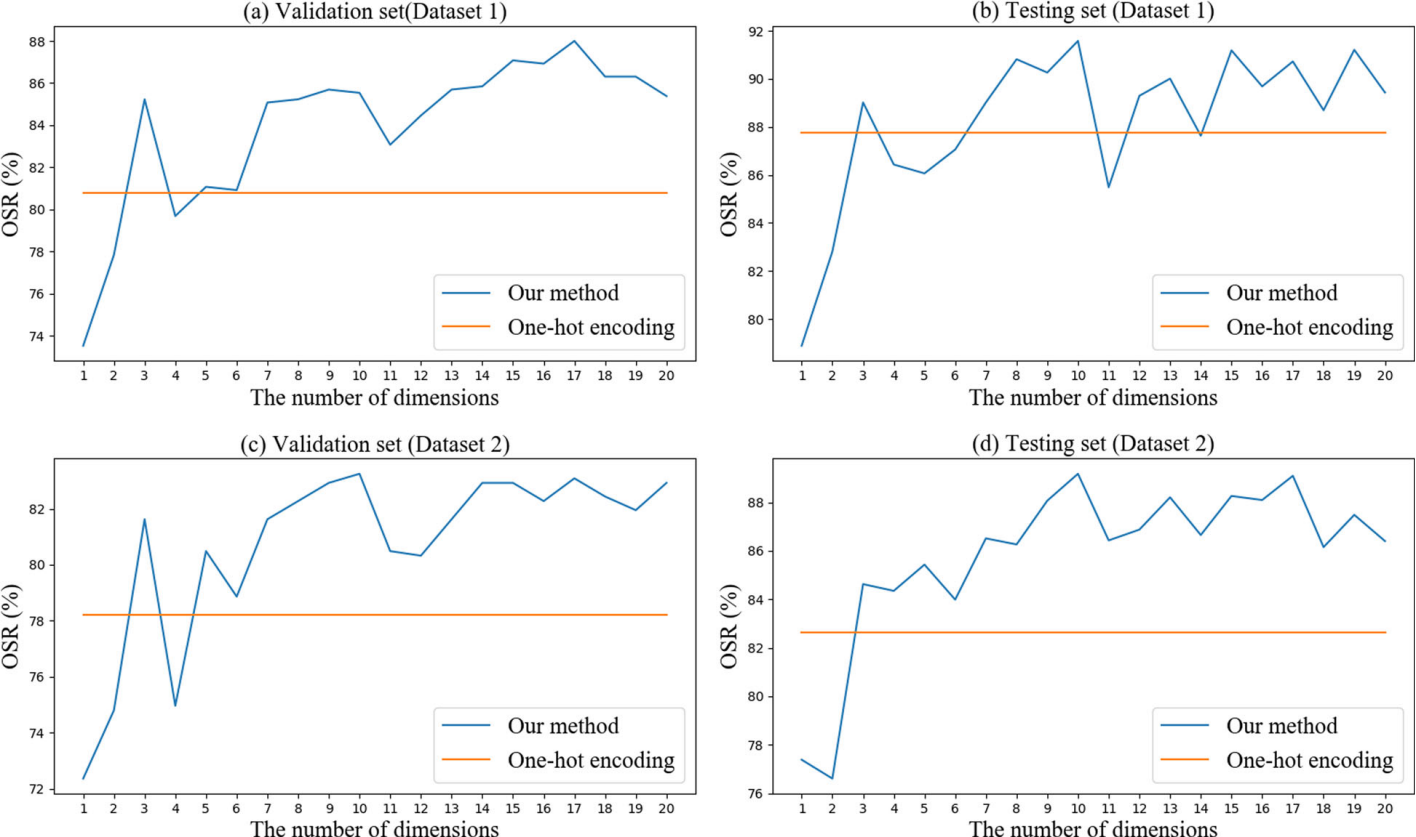
Comparison of the overall success rate between our method and one-hot encoding on Dataset 1 and Dataset 2. The x-axis represents the number of dimensions reduced by the autoencoder. The y-axis represents the overall success rate. **a** and **b** show the results of the validation set and testing set on Dataset 1; **c** and **d** show the results on Dataset 2

Figure 6 shows the overall success rate of vector representations using different dimensions in Dataset 1 and Dataset 2. To verify whether the vector representation that we proposed is better than one-hot encoding, we also indicated the overall success rate of the one-hot encoding method in the figures. It can be seen from the figure that our method is better in most cases. Taking both the prediction performance and computational complexity into account, we finally chose the vector representation with 10-dimension to replace the one-hot encoding.

Furthermore, to verify whether the vector representation is helpful to improve the performance of the model, we also randomly generate a 10-dimensional vector in the interval [0,1] for comparison experiments; the results are shown in Table 3. From the table, we find that the vector representation that we proposed is more effective than other methods both on Dataset 1 and Dataset 2. In addition, we find that the random method is even worse than the one-hot encoding. Although the random method has a lower dimension, it cannot express the relationship between amino acids well, which makes the result worse. The final experimental results illustrate the reliability of the new vector representation method that we proposed.

**Table 3:** Comparison of the overall success rate between different vector representation methods on Dataset 1 and Dataset 2

Method	Dataset 1		Dataset 2	
	Validation set	Testing set	Validation set	Testing set
One hot encoding	80.78%	87.77%	78.21%	82.63%
Random method	74.77%	80.87%	71.22%	81.94%
Our method	**85.54%**	**91.58%**	**83.25%**	**89.18%**

Note: Best performing method in bold.

### The effective of sequence information model

Table 4 gives the overall success rate of the comparison between the sequence information model and traditional models on Dataset 1 and Dataset 2, where four popular features used for comparative experiments are AAC, DipC, TipC and PseAAC. Among them, AAC, DipC and Tipc are amino acid composition-based methods; PseAAC is the method based on amino acid composition and physicochemical properties. These features are all extracted from the membrane protein sequence. The machine learning algorithm used to compare random forests. Research has illustrated its effectiveness in the prediction of membrane protein types [50]. The optimal parameters in random forests are obtained by using the grid search method and 5-fold cross-validation on the training set. From the table, we find that our DNN model has a better overall success rate than all the machine learning methods. Compared with the DipC, which has the best results among the four sequence features, our model improved by 5.43% and 8.30% on Dataset 1 and Dataset 2, respectively.

**Table 4:** Comparison of the overall success rate between four traditional models and sequence information model on Dataset 1 and Dataset 2

Method	Dataset 1	Dataset 2
	Overall success rate	Overall success rate
AAC+RF	85.02%	79.55%
DipC+RF	86.12%	80.88%
TipC+RF	84.21%	79.00%
PseAAC+RF	85.74%	80.27%
Our model	**91.58%**	**89.18%**

Note: Best performing method in bold.

Tables 5 and 6 give the values of Se, Sp, ACC and MCC for eight membrane protein types using four traditional models and our DNN model on Dataset 1 and Dataset 2, respectively. Although our model is not as good as the traditional model in some indicators, our model achieves better results in most cases. Furthermore, we find that it is difficult for the method combining feature engineering and the machine learning algorithm to predict the small types, especially in Type 4 membrane protein samples where the value of Sn and Mcc is zero in most traditional models. However, our model can predict the small type samples well. The reason may be the powerful feature extraction capabilities of the convolutional layer and Bi-LSTM layer that we used. The experimental results prove that our proposed sequence information model has better performance than traditional models.

**Table 5:** Comparison of the Se, Sp, ACC and MCC between four traditional models and sequence information model on Dataset 1

Membrane protein types	Index	AAC	DipC	TipC	PseAAC	Our model
	Se	0.7928	0.7095	0.5225	0.8221	**0.8356**
Type I	Sp	0.9537	0.9671	0.9805	0.9568	**0.9941**
	ACC	0.9372	0.9407	0.9335	0.9430	**0.9778**
	MCC	0.6898	0.6772	0.5936	0.7192	**0.8751**
	Se	0.2692	0.0641	0.0256	0.2949	**0.8077**
Type II	Sp	0.9946	**0.9993**	1.0	0.9951	0.9871
	ACC	0.9815	0.9825	0.9825	0.9825	**0.9838**
	MCC	0.3499	0.1964	0.1587	0.3845	**0.6492**
	Se	0.0	0.0	0.0	0.0	**0.1667**
Type III	Sp	**1.0**	**1.0**	**1.0**	**1.0**	0.9998
	ACC	**0.9986**	**0.9986**	**0.9986**	**0.9986**	**0.9986**
	MCC	0.0	0.0	0.0	0.0	**0.2881**
	Se	0.0833	0.0	0.0	0.0	**0.6667**
Type IV	Sp	**1.0**	**1.0**	**1.0**	**1.0**	0.9984
	ACC	0.9975	0.9972	0.9972	0.9972	**0.9986**
	MCC	0.2883	0.0	0.0	0.0	**0.5950**
	Se	0.9069	0.9587	**0.9816**	0.9029	0.9461
Mutipass	Sp	0.8661	0.7004	0.4766	0.8858	**0.9092**
	ACC	0.8968	0.8950	0.8571	0.8987	**0.9370**
	MCC	0.7390	0.7051	0.5847	0.7479	**0.8355**
	Se	0.1579	0.0789	0.0263	**0.1579**	**0.1579**
Lipid-chain-anchored	Sp	0.9988	**1.0**	**1.0**	0.9993	0.9979
	ACC	0.9915	**0.9919**	0.9915	**0.9919**	0.9905
	MCC	0.2904	0.2798	0.1615	**0.3219**	0.2473
	Se	0.3696	0.0435	0.0435	0.3478	**0.8478**
GPI-anchored	Sp	0.9974	**1.0**	**1.0**	0.9960	0.9951
	ACC	0.9908	0.9898	0.9898	0.9892	**0.9935**
	MCC	0.4694	0.2075	0.2075	0.4054	**0.7393**
	Se	0.7342	0.6261	0.4662	0.8041	**0.8806**
Peripheral	Sp	0.9262	0.9617	**0.9874**	0.9262	0.9609
	ACC	0.9065	0.9273	0.9340	0.9137	**0.9527**
	MCC	0.5747	0.5981	0.5835	0.6226	**0.7708**

Note: Best performing method in bold.

**Table 6:** Comparison of the Se, Sp, ACC and MCC between four traditional models and sequence information model on Dataset 2

Membrane protein types	Index	AAC	DipC	TipC	PseAAC	Our model
	Se	0.6571	0.5306	0.3388	0.6898	**0.8408**
Type I	Sp	0.9384	0.9643	**0.9824**	0.9440	0.9806
	ACC	0.9193	0.9348	0.9387	0.9267	**0.9711**
	MCC	0.4951	0.4903	0.4156	0.5341	**0.7841**
	Se	0.1139	0.0	0.0	0.0633	**0.7468**
Type II	Sp	0.9901	**1.0**	**1.0**	0.9889	0.9739
	ACC	0.9709	**0.9781**	**0.9781**	0.9686	0.9689
	MCC	0.1387	0.0	0.0	0.0696	**0.5267**
	Se	0.0	0.0	0.0	0.0	0.0
Type III	Sp	**1.0**	**1.0**	**1.0**	**1.0**	**1.0**
	ACC	**0.9975**	**0.9975**	**0.9975**	**0.9975**	**0.9975**
	MCC	0.0	0.0	0.0	0.0	0.0
	Se	0.0	0.0	0.0	0.0	**0.7647**
Type IV	Sp	**1.0**	**1.0**	**1.0**	**1.0**	0.9958
	ACC	**0.9953**	**0.9953**	**0.9953**	**0.9953**	0.9947
	MCC	0.0	0.0	0.0	0.0	**0.5935**
	Se	0.8721	0.9290	**0.9617**	0.8709	0.9157
Mutipass	Sp	0.8526	0.7105	0.4885	0.8588	**0.9813**
	ACC	0.8660	0.8607	0.8138	0.8671	**0.9362**
	MCC	0.7022	0.6669	0.5442	0.7058	**0.8638**
	Se	**0.0556**	0.0	0.0	0.0278	0.0
Lipid-chain-anchored	Sp	**1.0**	**1.0**	**1.0**	**1.0**	0.9983
	ACC	**0.9906**	0.9900	0.9900	0.9903	0.9883
	MCC	**0.2346**	0.0	0.0	0.1659	-0.0041
	Se	0.3659	0.0488	0.0	0.3659	**0.9024**
GPI-anchored	Sp	0.9986	**1.0**	**1.0**	0.9941	0.9938
	ACC	0.9914	0.9892	0.9886	0.9870	**0.9928**
	MCC	0.5203	0.2197	0.0	0.3839	**0.7490**
	Se	0.7425	0.6881	0.5451	0.7797	**0.9013**
Peripheral	Sp	0.8885	0.9164	**0.9580**	0.8954	0.9418
	ACC	0.8602	0.8721	0.8779	0.8729	**0.9340**
	MCC	0.5893	0.5965	0.5740	0.6290	**0.8025**

Note: Best performing method in bold.

### The effective of evolutionary information model

Then, we use the same experimental method to compare the performance between the evolutionary information model and seven popular PSSM-based tradition methods proposed by other researchers, including EDPSSM, KPSSM, CPSSM, TriPSSM, RPSSM, CoPSSM and PsePSSM. They are all powerful features extracted from PSSM. Table 7 gives the overall success rate between seven traditional models and the evolutionary information model on Dataset1 and Dataset 2. From the table, we find the best method for the traditional model is TriPSSM, which utilizes PSSM linear probabilities to compute features. Although TriPSSM can obtain the outstanding grades on membrane protein types prediction, we find that it has 8000-dimensional features; the high dimensionality of features in turn increases the computing complexity. Our model is still effective since it can use GPU acceleration to yield better results in less time.

**Table 7:** Comparison of the overall success rate between seven traditional models and evolutionary information model on Dataset 1 and Dataset 2, respectively

Method	Dataset 1	Dataset 2
	Overall success rate	Overall success rate
EDPPSSM+RF	86.82%	79.83%
KPSSM+RF	92.34%	87.99%
CPSSM+RF	88.83%	83.55%
TriPSSM+RF	93.21%	89.43%
RPSSM+RF	90.56%	84.05%
CoPSSM+RF	90.79%	84.21%
PsePSSM+RF	91.71%	86.85%
Our model	**94.02%**	**89.79%**

Note: Best performing method in bold.

Tables 8 and 9 report the results, measured in Se, Sp, ACC and MCC over all methods on Dataset 1 and Dataset 2. From the tables, we can draw a conclusion that our model is more effective than the traditional model in evolution information extraction. The convolutional layer has a powerful ability to extract features from the evolutionary information, and the CapsNet layer can extract local position information. These abilities explain why our DNN model could achieve better results.

**Table 8:** Comparison of the Se, Sp, ACC and MCC between seven traditional models and the evolutionary information model on Dataset 1

Membrane protein types	Index	PsePSSM	RPSSM	EDPSSM	KPSSM	CPSSM	CoPSSM	TriPSSM	Our model
	Se	0.9122	0.8761	0.8491	0.9054	0.8739	0.8806	0.9122	**0.9144**
Type I	Sp	0.9776	0.9733	0.9622	0.9859	0.9666	0.9781	0.9884	**0.9920**
	ACC	0.9709	0.9633	0.9506	0.9776	0.9571	0.9682	0.9806	**0.9841**
	MCC	0.8505	0.8112	0.7546	0.8800	0.7856	0.8328	0.8953	**0.9129**
	Se	0.7308	0.6410	0.5256	0.6923	0.4231	0.6923	0.8205	**0.8462**
Type II	Sp	0.9927	0.9918	0.9960	0.9913	**0.9979**	0.9922	0.9929	0.9944
	ACC	0.9880	0.9855	0.9875	0.9859	0.9875	0.9868	0.9898	**0.9912**
	MCC	0.6819	0.6067	0.6035	0.6339	0.5713	0.6489	0.7424	**0.7748**
	Se	**0.1667**	0.0	0.0	**0.1667**	0.0	0.0	**0.1667**	**0.1667**
Type III	Sp	**1.0**	0.9998	**1.0**	**1.0**	**1.0**	**1.0**	**1.0**	0.9993
	ACC	**0.9988**	0.9984	0.9986	**0.9988**	0.9986	0.9986	**0.9988**	0.9984
	MCC	**0.4080**	-0.0006	0.0	0.4080	0.0	0.0	**0.4080**	0.2032
	Se	0.4167	0.5000	0.4167	0.6667	0.0833	0.3333	0.6667	**0.75**
Type IV	Sp	0.9995	0.9993	**1.0**	0.9995	**1.0**	0.9998	0.9995	0.9995
	ACC	0.9979	0.9979	0.9984	**0.9986**	0.9975	0.9979	**0.9986**	0.9982
	MCC	0.5446	0.5763	0.6450	0.7296	0.2883	0.5156	0.7296	**0.7828**
	Se	0.9275	0.9394	0.9219	0.9495	0.9305	0.9326	0.9553	**0.9593**
Mutipass	Sp	0.9654	0.9438	0.8305	0.9625	0.9026	0.9682	0.9691	**0.9785**
	ACC	0.9444	0.9405	0.8994	0.9527	0.9236	0.9414	0.9587	**0.9640**
	MCC	0.8619	0.8493	0.7360	0.8799	0.8042	0.8560	0.8947	**0.9083**
	Se	0.3158	0.2368	0.2105	0.3684	0.2105	0.3684	0.3158	**0.6053**
Lipid-chain-anchored	Sp	0.9986	0.9979	0.9977	0.9984	**0.9991**	0.9972	0.9970	0.9960
	ACC	0.9926	0.9912	0.9908	**0.9928**	0.9922	0.9917	0.9910	0.9926
	MCC	0.4557	0.3403	0.3018	0.4924	0.3719	0.4414	0.3850	**0.5862**
	Se	0.6522	0.5217	0.3913	0.6957	0.3913	0.7174	0.6957	**0.8043**
GPI-anchored	Sp	0.9963	0.9986	0.9967	0.9986	**0.9988**	0.9951	0.9984	0.9963
	ACC	0.9926	0.9935	0.9903	**0.9954**	0.9924	0.9922	0.9952	0.9942
	MCC	0.6484	0.6431	0.4645	0.7631	0.5503	0.6582	**0.7531**	0.7465
	Se	**0.9077**	0.8536	0.6824	0.8784	0.8176	0.8851	0.8964	0.9009
Peripheral	Sp	0.9537	0.9509	0.9481	0.9524	0.9403	0.9452	0.9578	**0.9637**
	ACC	0.9490	0.9409	0.9208	0.9448	0.9278	0.9391	0.9515	**0.9573**
	MCC	0.7655	0.7218	0.5959	0.7427	0.6678	0.7260	0.7711	**0.7933**

Note: Best performing method in bold.

**Table 9:** Comparison of the Se, Sp, ACC and MCC between seven traditional models and the evolutionary information model on Dataset 2

Membrane protein types	Index	PsePSSM	RPSSM	EDPSSM	KPSSM	CPSSM	CoPSSM	TPSSM	Our model
	Se	**0.8571**	0.7633	0.7265	0.7959	0.7633	0.7673	0.8653	0.8082
Type I	Sp	0.9586	0.9574	0.9512	0.9705	0.9556	0.9550	0.9759	**0.9845**
	ACC	0.9517	0.9442	0.9359	0.9587	0.9426	0.9423	0.9684	**0.9725**
	MCC	0.6943	0.6290	0.5821	0.7048	0.6224	0.6229	0.7748	**0.7853**
	Se	0.4304	0.4051	0.3038	**0.4810**	0.2785	0.4304	0.4557	0.4684
Type II	Sp	0.9887	0.9881	0.9904	0.9889	**0.9952**	0.0.9858	0.9906	0.9901
	ACC	0.9764	0.9753	0.9753	0.9778	**0.9795**	0.9736	0.9789	0.9786
	MCC	0.4327	0.4059	0.3423	0.4759	0.3873	0.4039	0.4769	**0.4797**
	Se	0.0	0.0	0.0	0.0	0.0	0.0	0.0	**0.1111**
Type III	Sp	**1.0**	**1.0**	**1.0**	**1.0**	**1.0**	**1.0**	**1.0**	0.9958
	ACC	**0.9975**	**0.9975**	**0.9975**	**0.9975**	**0.9975**	**0.9975**	**0.9975**	0.9936
	MCC	0.0	0.0	0.0	0.0	0.0	0.0	0.0	**0.0803**
	Se	0.1765	0.1176	0.0588	**0.3529**	0.1176	0.2353	**0.3529**	**0.3529**
Type IV	Sp	0.9994	0.9997	**1.0**	**1.0**	**1.0**	**1.0**	**1.0**	0.9980
	ACC	0.9956	0.9956	0.9956	**0.9969**	0.9958	0.9964	**0.9969**	0.9950
	MCC	0.3238	0.2788	0.2420	**0.5932**	0.3423	0.4842	**0.5932**	0.4011
	Se	0.9044	0.8971	0.8882	0.9350	0.9023	0.8910	0.9435	**0.9443**
Mutipass	Sp	0.9494	0.9050	0.7655	0.9263	0.8712	0.9334	0.9316	**0.9574**
	ACC	0.9184	0.8996	0.8499	0.9323	0.8926	0.9043	0.9398	**0.9484**
	MCC	0.8232	0.7776	0.6517	0.8464	0.7572	0.7930	0.8627	**0.8839**
	Se	0.1111	0.0278	0.0833	0.0833	0.0278	0.0833	0.0833	**0.6111**
Lipid-chain-anchored	Sp	0.9986	**0.9997**	**0.9997**	0.9986	0.9994	0.9992	0.9983	0.9927
	ACC	0.9897	0.9900	**0.9906**	0.9895	0.9897	0.9900	0.9892	0.9889
	MCC	0.2186	0.1161	0.2480	0.1731	0.0939	0.2012	0.1627	**0.5238**
	Se	0.4146	0.2927	0.2439	0.4146	0.2927	0.3902	0.4146	**0.7073**
GPI-anchored	Sp	0.9961	0.9986	0.9983	0.9949	**0.9992**	0.9983	**0.9992**	0.9966
	ACC	0.9895	0.9906	0.9897	0.9914	0.9911	0.9881	0.9925	**0.9933**
	MCC	0.4717	0.4508	0.3864	0.5381	0.4807	0.4226	0.5907	**0.7039**
	Se	**0.8884**	0.8183	0.6581	0.8512	0.7883	0.8326	0.8741	0.8627
Peripheral	Sp	0.9253	0.9050	0.9112	0.9312	0.9046	0.9064	0.9377	**0.9404**
	ACC	0.9181	0.8882	0.8621	0.9156	0.8821	0.8921	**0.9254**	**0.9254**
	MCC	0.7616	0.6742	0.5635	0.7460	0.6513	0.6872	**0.7752**	0.7725

Note: Best performing method in bold.

### The effective of stacking ensemble method

To investigate on how the fusion model impacts the performance, we report the overall success rate of the validation set and testing set using two single models and the fusion model (Table 10). From the table, we find that the fusion model achieves better results both on datasets, demonstrating its superiority in prediction.

**Table 10:** Comparison of the overall success rate between two single information model and fusion model on Dataset 1 and Dataset 2

Method	Dataset 1		Dataset 2	
	Validation set	Testing set	Validation set	Testing set
Sequence information model	85.54%	91.58%	83.25%	89.18%
Evolutionary information model	90.46%	94.02%	80.98%	89.79%
Fusion model	—	**95.68%**	—	**92.98%**

Note: Best performing method in bold.

**Fig. 7:**
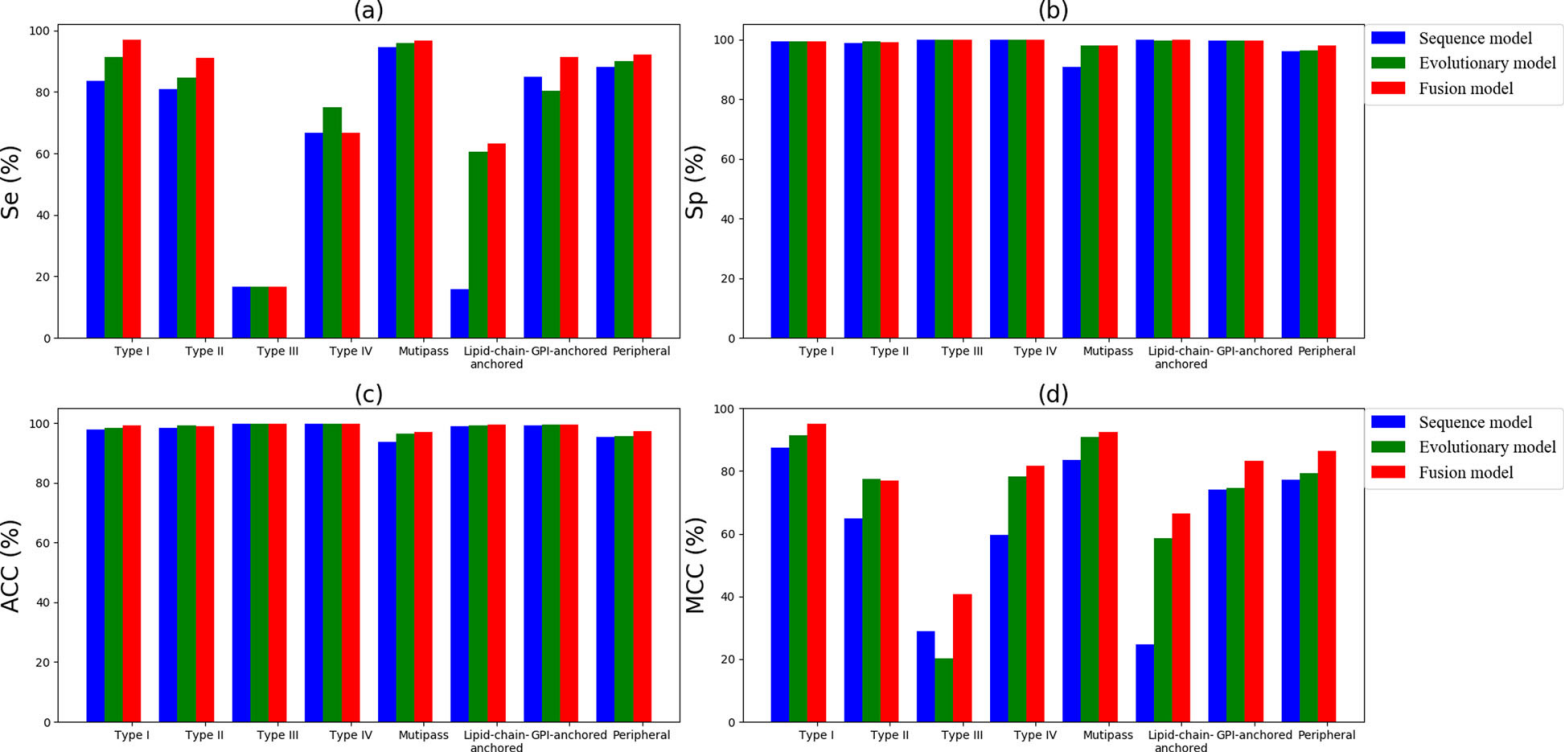
Comparison of two single models with the fusion model on Dataset 1: **a** Sensitivity (Se); **b** Specificity (Sp); **c** Accuracy (ACC) and **d** Mathews correlation coeffcient (MCC)

**Fig. 8:**
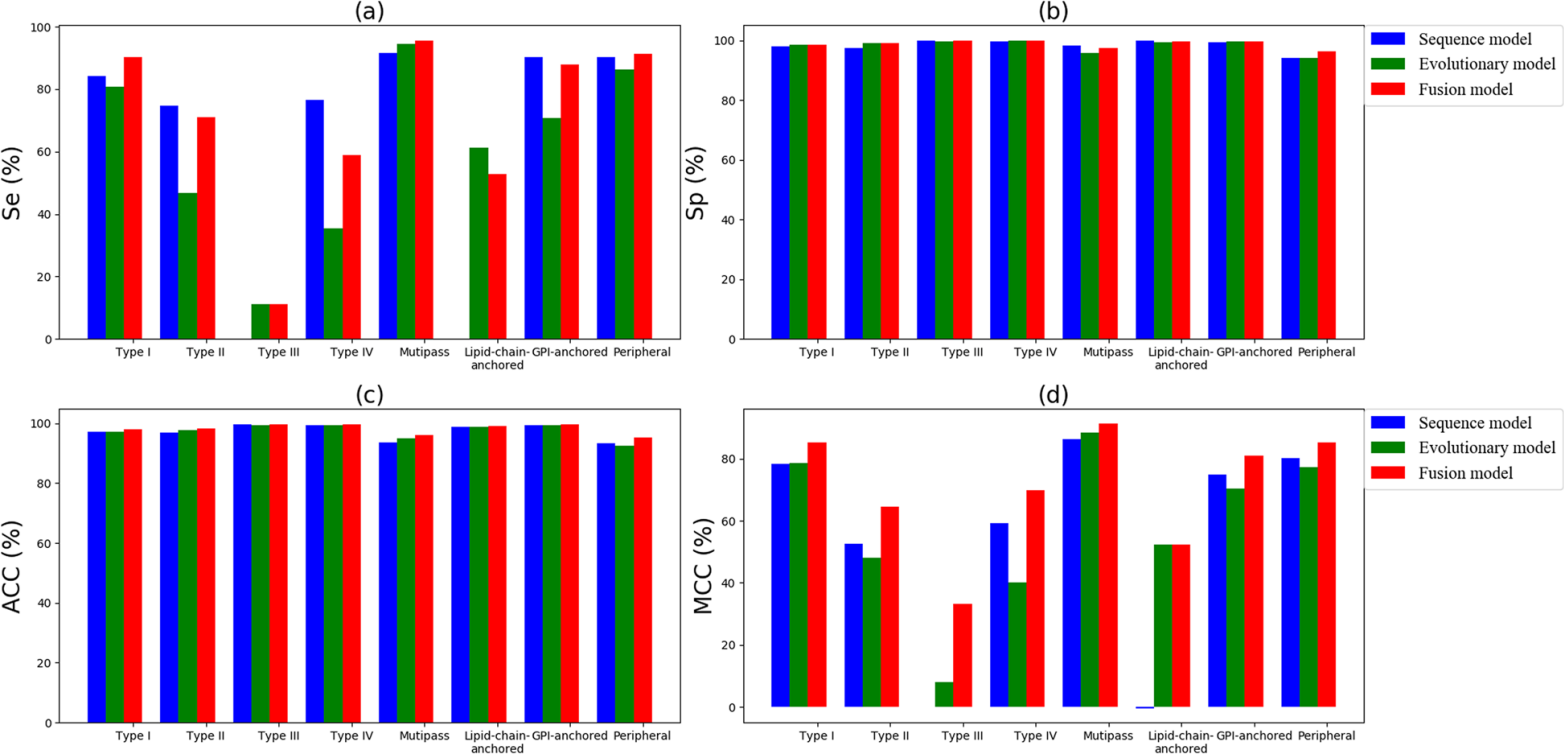
Comparison of two single models with the fusion model on Dataset 2: **a** Sensitivity (Se); **b** Specificity (Sp); **c** Accuracy (ACC) and **d** Mathews correlation coeffcient (MCC)

Figures 7 and 8 give the comparison results, measured in Se, Sp, ACC and MCC over three models on Dataset 1 and Dataset 2,respectively. We find that the fusion model achieves the best results in most cases. However, the performance of small-scale forecasts has not yet met our expectations. Other effective strategies for solving imbalance problems may contribute to improving our method.

Furthermore, the prediction of membrane protein types requires not only high classification accuracy but also certain operational efficiency. Table 11 lists the running time for different models when predicting 100 membrane protein samples. We find that the process of fusion takes approximately 1.93e-10s, and the running time of the fusion model is almost the sum of two single models. Though the time complexity of the fusion model is higher, it is also within the tolerance range.

**Table 11:** Comparison of the running time for different models when predicting 100 membrane protein samples

Model	Running time (s)
Sequence information model	0.97
Evolutionary information model	0.50
Fusion model	1.47

### Comparison with the existing methods

To demonstrate the performance of our DNN model in practical use, we compared our DNN model with eight state-of-the-art machine learning methods in the prediction of membrane protein types. These models all proceed through complex feature engineering, including feature extraction, dimension reduction and so on. Table 12 represents the overall success rate between our DNN model with eight models on Dataset 1 and Dataset 2. Taking the accuracy of prediction and the degree of domain experts dependence into account, we find the DNN model not only achieves the best performance both on Dataset 1 and Dataset 2, but also removes the burden of feature engineering, which illustrates the effectiveness of our model.

**Table 12:** Comparison of the overall success rate with eight state-of-the-art methods

Method	Overall success rate	
	Dataset 1	Dataset 2
AAC based on Covariance [9]	37.2%	—
PsePSSM based on ensemble method [9]	91.6%	78.3%
Physicochemical properties based on ensemble method [13]	91.0%	—
Fusion representation based on SVM [16]	92.6%	88.2%
PsePSSM based on LLDA and ensemble method [22]	88.7%	—
PsePSSM and DC based on GPP and KNN [20]	90.2%	—
PsePSSM based on PCA and KNN [22]	80.66%	—
Previous work [19]	93.49%	—
This paper	**95.68%**	**92.98%**

Note: Best performing method in bold.

## Conclusion

In this paper, we propose two DNN models to process the sequence information and evolutionary information separately. Among these models, 1D convolutional layers and Bi-LSTM layers are used for the sequence information model, while the evolutionary information model uses 2D convolutional layers and CapsNet layers. In the comparative experiments, we compare these models with the traditional model. Numerous experimental results demonstrate that our two proposed DNN models not only obtain more competitive results in membrane protein type prediction but also can remove the burden of feature engineering and the reliance on domain experts. In addition, to obtain a better prediction performance, the stacking ensemble method is used to fuse the above two models.

Furthermore, we provide a new vector representation method based on autoencoder and the physicochemical properties of amino acids to improve the performance of the sequence information model. Compared with the one-hot encoding, the new vector representation method not only represents the relationship between amino acids well but also can effectively improve the prediction performance.

Finally, our method can be applied not only to membrane protein type prediction but also to other fields of bioinformatics [51–63]. However, there is still room for further investigation. For example, the problem of an imbalanced dataset had a negative effect on the accuracy of small-sized types. Some strategies for processing with the imbalanced dataset may improve the performance of our DNN model.

## Data Availability

The related source codes and datasets are available at https://github.com/DragonKnightss/MembraneProteinTypePrediction.

## Abbreviations

ACC: accuracy
AD: autocorrelation descriptor
Bi-LSTM: bidirectional long short term memory
CBOW: Continuous bag of words
CoPSSM: correlation position-specific scoring matrix
DipC: dipeptide composition
DNN: deep neural network
EDPSSM: evolutionary difference position-specific score matrix
FN: false negative
FP: false positive
GD: global descriptor
HHT: Hilbert-Huang transform
KNN: k-nearest neighbor
KPSSM: k-separated-bigrams position-specific score matrix
LAAC: Local amino acid composition
LDC: local dipeptide composition
LZC: Lempel-Ziv complexity
MCC: Matthews correlation coefficient
MLR: multiple logistic regression. NBM: naive bayesian model
NLP: natural language processing
OSR: overall success rate
PNN: probabilistic neural network
PseAAC: pseudo-amino acid composition
PsePSSM: pseudo position-specific score matrix
PSSM: position-specific scoring matrix
RF: random forests
RPSSM: reduced position-specific score matrix
SD: sequence-order descriptor
Se: sensitivity
Sp: specificity
SVM: support vector machines
TipC: tripeptide composition
TN: true negative
TP: true positive
TriPSSM: tri-gram position-specific score matrix
